# Phenotypic and Molecular Selection of a Superior *Solanum pennellii* Introgression Sub-Line Suitable for Improving Quality Traits of Cultivated Tomatoes

**DOI:** 10.3389/fpls.2019.00190

**Published:** 2019-02-22

**Authors:** Roberta Calafiore, Antonietta Aliberti, Valentino Ruggieri, Fabrizio Olivieri, Maria Manuela Rigano, Amalia Barone

**Affiliations:** Department of Agricultural Sciences, University of Naples Federico II, Portici, Italy

**Keywords:** *S. lycopersicum*, production traits, fruit quality traits, ascorbic acid, firmness, molecular markers, candidate gene identification

## Abstract

The *Solanum pennellii* Introgression Line (IL) population can be exploited to identify favorable alleles that can improve yield and fruit quality traits in commercial tomato varieties. Over the past few years, we have selected ILs that exhibit increased content of antioxidant compounds in the fruit compared to the cultivar M82, which represents the genetic background in which the different wild regions of the *S. pennellii* ILs were included. Recently, we have identified seven sub-lines of the IL7-3 accumulating different amounts of antioxidants in the ripe fruit. Since the wild region carried on chromosome 7 induces a low fruit production in IL7-3, the first aim of the present work was to evaluate yield performances of the selected sub-lines in three experimental fields located in the South of Italy. Another aim was to confirm in the same lines the high levels of antioxidants and evaluate other fruit quality traits. On red ripe fruit, the levels of soluble solids content, firmness, and ascorbic acid (AsA) were highly variable among the sub-lines grown in three environmental conditions, evidencing a significant genotype by environment interaction for soluble solids and AsA content. Only one sub-line (coded R182) exhibited a significantly higher firmness, even though no differences were observed for this trait between the parental lines M82 and IL7-3. The same sub-line showed significantly higher AsA content compared to M82, thus resembling IL7-3. Even though IL7-3 always exhibited a significantly lower yield, all the sub-lines showed yield variability over the three trials. Interestingly, the sub-line R182, selected for its better performances in terms of fruit quality, in all the trials showed a production comparable to that of the control line M82. A group of species-specific molecular markers was tested on R182 and on the parental genotypes in order to better define the wild genomic regions carried by the elite line R182. In these regions three candidate genes that could increase the level of AsA in the fruit were identified. In the future, the line R182 could be used as pre-breeding material in order to obtain new varieties improved for nutritional traits.

## Introduction

Tomato (*Solanum lycopersicum* L.) is one of the most important and most extensively used crops worldwide and a major component of the Mediterranean diet, even if fruit quality and flavor have deteriorated in recent years (Liu et al., 2016; Rambla et al., 2017). Indeed, the tomato commercial varieties used today are the result of several rounds of domestication and intense breeding activities and contain lower amounts of flavor chemicals compared to older varieties (Fernandez-Moreno et al., 2017; Tieman et al., 2017). This is also the result of the breeding efforts that have been initially focused mostly on yield, disease resistance, and firmness (Ballester et al., 2016). For these reasons, in the last two decades fruits nutritional and organoleptic quality, including content of antioxidants, flavor and Brix degree are becoming major targets of tomato breeders (Sacco et al., 2013; Tieman et al., 2017). The natural variation existing in the 12 known wild tomato relatives is a potential source of unused beneficial alleles that were lost during the process of domestication and that could be used for the improvement of the cultivated tomato varieties (Gur et al., 2011; Liu et al., 2016). In this regard, powerful genetic resources consist of introgression lines (ILs) populations obtained from wild species including *Solanum pennellii*, *Solanum habrochaites*, *Solanum lycopersicoides*, *Solanum pimpinellifolium* (Eshed and Zamir, 1995; Fridman et al., 2004; Canady et al., 2005; Barrantes et al., 2016). In particular, the *Solanum pennellii* ILs population comprises a core set of 76 lines that contain marker-defined overlapping chromosomal segments of the wild genome in the background of the cultivated variety M82 (Alseekh et al., 2013; Krause et al., 2018). In several studies, the use of these ILs allowed the identification of loci connected to a number of phenotypes by associating the phenotypic variations detected in different ILs to the introgression segments (Lippman et al., 2007). In particular, the *S. pennellii* ILs population has been previously used to identify a number (more than 3000) of QTLs (quantitative trait loci) affecting morphology, stress tolerance, plant yield, fruit color and metabolism (Eshed and Zamir, 1995; Alseekh et al., 2013; Krause et al., 2018). In the last few years, in our and in other laboratories this genetic material has been used to identify genes and alleles controlling antioxidants production and accumulation in tomato fruit and to produce superior genetic materials with increased amounts of bioactive compounds in the fruit (Causse et al., 2004; Sacco et al., 2013; Rigano et al., 2014). In addition, novel genetic resources are now available, including a new backcrossed inbred line (BIL) population that was generated by repeated backcrossing of a F_1_ hybrid between the cultivated line M82 and *S. pennellii* and its progeny to M82, followed by selfing (Ofner et al., 2016). This BIL population could be used in combination with ILs for fine-mapping QTLs previously identified and to pinpoint strong candidate genes (Fulop et al., 2016). Moreover, the *Solanum pennellii* ILs have been broken into additional sub-lines carrying molecular marker-defined introgressions that are smaller than those carried by the original ILs, further facilitating the identification of candidate genes (Alseekh et al., 2013). These sub-isogenic lines are now available to the scientific community and have been recently used to map loci affecting fruit chemical composition (Alseekh et al., 2015; Liu et al., 2016).

We previously identified one *S. pennellii* IL (IL7-3) carrying a wild region on chromosome 7 and exhibiting high levels of antioxidants and °Brix in the fruit (Di Matteo et al., 2010, 2013). However, this line was reported to carry a negative QTL for yield (Eshed and Zamir, 1995; Eshed et al., 1996; Sacco et al., 2013). Afterwards, we obtained seven sub-lines from IL7-3 and defined their wild region sizes by using molecular markers (Calafiore et al., 2016). The aims of the present work were (1) to select one or more superior sub-lines that combine favorable alleles/traits deriving from both the parental lines *S. lycopersicum* cv. M82 and IL7-3, and (2) to understand the effect of the wild alleles mapping in the region IL7-3 on yield and fruit quality traits. Various fruit quality traits and yield performances were here analyzed in the seven different IL7-3 sub-lines in three different experimental fields located in the South of Italy and in two different years. These analyses allowed us to identify one elite sub-line with improved qualitative traits. A group of gene-targeting markers was also tested on the selected sub-line and the parental genotypes in order to better define the wild genomic regions carried by the elite line. Altogether, this work allowed us to identify the genomic region that has major effects on different quality traits and to select one superior genotype that could be used in breeding schemes in order to obtain new varieties with improved nutritional traits, such as higher ascorbic acid (AsA) content in mature fruit.

## Materials and Methods

### Plant Material

Plant material consisted of the cultivated genotype M82 (LA3475), the *S. pennellii* in *S. lycopersicum* IL7-3 (accession LA4102), previously selected in our laboratory for high content of AsA in the fruit (Di Matteo et al., 2010), and seven sub-lines of the region 7-3 previously obtained and characterized as described in Calafiore et al. (2016). The purity and stability of the parental IL7-3 was tested by Long et al. (2013) using more than 800 SSR markers distributed all over the tomato genome. Also in our laboratory, the stability of IL7-3 was established by screening more than 7000 SNP markers of the SolCAP genomic platform (data not shown). From IL7-3 two sub-lines (coded R201 and R202) were obtained in our laboratory (Calafiore et al., 2016), and five sub-lines (coded R176-R182) were kindly provided by Dr. Dani Zamir (Hebrew University, Israel). Crosses and self-generations to get the sub-lines were obtained under strictly controlled conditions by using mosquito netting and blossom bags.

For two years (2016-2017), plants at a four leaf-stage were transplanted in open field in three different locations using single rows with plant spacing of 40 cm × 50 cm. Soil texture was classified as sandy-loam in all the experimental fields and details about soil composition are reported in Supplementary Table S1. Urea phosphate fertilizer (40 kg ha^−1^) was applied to the soil before transplanting. Plowing followed by one or two milling were used as tillage treatments. In absence of rain plants were irrigated as required (2-3 times per week). Levels of N (190 kg ha^−1^), P (25 kg ha^−1^), and K (20 kg ha^−1^) were maintained during cultivation *via* fertirrigation.

Data collected in the year 2016 are related to one field (Acerra, Campania Region), whereas in the year 2017 data were collected from plants grown in three fields located in the Campania Region (Acerra, Battipaglia, and Giugliano). A randomized complete block design with three replicates *per* genotype and 10 plants *per* replicate was assessed in all the fields. Three plants *per* genotype were used for measuring traits related to fruit set (FS) and number of flowers/inflorescence (NFL). Data recorded as number of fruit *per* plant (NFR), fruit weight (FW), and yield *per* plant (YP) were collected from all the plants of each genotype.

Tomato fruits were collected at mature red (MR) stage, seeds and columella were subsequently removed, and fruits were ground in liquid nitrogen and stored at −80°C until further analyses.

### Qualitative Analyses

For each genotype and biological replicate 15 fruits were collected at MR stage to evaluate soluble solids content, firmness, and color. The soluble solids content was measured as °Brix in the homogenized juice from ripe fruit by a refractometer (Hanna), the firmness (FI) of fruit cuticle was measured on ripe fruit by a penetrometer with an 8 mm shore (PCE-PTR200 penetrometer). The color of ripe fruit was assessed as percentage of reflectance (L) and absorbance index (a/b), where a is the absorbance at 540 nm and b at 675 nm, using a Konica Minolta CR-400a, and performing two measures *per* ripe fruit (six fruits *per* genotype). The software Tomato Analyzer 3.0^1^ (Rodríguez et al., 2010) was used for the fruit morphological characterization using approximately six fruits *per* genotype to measure the fruit perimeter (FP), fruit area (FA), pericarp area (PA), pericarp thickness (PT), distal (DA), and proximal angles (micro PA and macro PA, respectively).

#### Ascorbic Acid Determination

A colorimetric method was used for the AsA determination (Stevens et al., 2006) with modifications reported by Rigano et al. (2014). Briefly, 300 μl of ice-cold 6% TCA was used for 500 mg of frozen tomato fruit powder, afterwards the mixture was vortexed for 15 min, incubated on ice and centrifuged at 14000 rpm for 20 min at 4°C. In an Eppendorf tube were placed 20 μl of supernatant with 20 μl of 0.4 M phosphate buffer (pH 7.4) and 10 μl of double distilled (dd) water. Then, 80 μl of color reagent solution were prepared by mixing solution A [31% H_3_PO_4_, 4.6% (w/v) TCA, and 0.6% (w/v) FeCl_3_] with solution B [4% 2,2′-dipyridil (w/v)]. The mixture was incubated at 37°C for 40 min and measured at 525 nm by a NanoPhotometer^TM^ (Implen). Three separated biological replicates for each sample and three technical assays for each biological repetition were measured. The concentration was expressed in nmol of AsA according to the standard curve, designed over a range of 0–70 nmol; the values were then converted into mg/100 g of fresh weight (FW).

#### Carotenoids Determination

The method reported by Zouari et al. (2014) was used for carotenoids extraction. A solution of acetone/hexane (40/60, v/v) was added to one gram of tomato fruit powder and incubated for 15 min at room temperature. The mixture was centrifuged at 4000 rpm for 10 min and the absorbance of the supernatant was measured at 663, 645, 505, and 453 nm. Total carotenoids, lycopene, and β-carotene were determined by the equation reported by Wellburn (1994). Results were expressed as mg/100 g FW. All biological replicates *per* sample were analyzed in triplicate.

### Statistical Analysis

Data analysis was conducted using the SPSS Software version 23. The Student’s *t*-test was conducted for qualitative and quantitative traits to verify if genotypes were statistically different from the parental line M82. A univariate ANOVA analysis was performed to detect the genotype by environment (GxE) interaction. In order to identify the genotypes with a desirable combination of traits, an evaluation index (EI) was calculated by assigning to each trait a positive (+1) or negative (−1) score depending on its significantly higher or lower value than the control M82. The EI was calculated for yield-related traits and fruit quality-related traits, separately.

### Molecular Marker Analysis

SCAR and CAPS markers were designed in order to estimate the size of the wild region present in the IL sub-line R182. Genomic DNA was extracted from young leaves collected from M82, IL7-3, and R182 using the ISOLATE II Plant DNA Kit (Bioline). Primers for the PCR amplification were designed based on polymorphisms detected in the IL7-3 introgression region between the *S. lycopersicum* (SL3.0 assembly and iTAG3.2 annotation) and the *S. pennellii* (v2 Assembly) genomes, by investigating the Genome Browser available in the Sol Genomics Network database^2^. PCR amplification was carried out in 50 μl reaction volume containing 50 ng DNA, 1X of My Taq reaction buffer, 1.0 mM primer and 1 U My Taq DNA polymerase (Bioline). For designing CAPS markers, restriction enzymes suitable to detect polymorphic SNPs between the fragments amplified were found using the tool CAPS Designer available at the Sol Genomics Network^2^. Amplified and restricted fragments were visualized on agarose gel at different concentrations depending on their expected size.

## Results

### Multi-Trait Phenotypic Evaluation

The cultivated line M82 and seven sub-lines of the *S. pennellii* IL7-3, previously obtained and characterized (Calafiore et al., 2016), were analyzed in the year 2016 for productivity, measuring five yield-related traits. Moreover, fruit quality and fruit morphology were evaluated in the same lines by measuring eight different traits related to fruit quality and by measuring seven different parameters by using the Tomato Analyzer software.

In order to understand the possible causes related to the reduced yield previously observed in IL7-3 (Eshed and Zamir, 1995; Sacco et al., 2013) compared to M82, we investigated in all the lines the NFL, the percentage of FS, the FW, the NFR, and the final YP (Table 1). No genotypes showed values of NFL significantly different from the cultivated line M82. Indeed, NFL varied from 5.1 (in M82) to 5.9 (in the sub-line R202) with a population mean of 5.4. As for FS, IL7-3 showed a significantly lower value than M82 (35.6% vs. 56.0%), with a reduction of approximately 36%. Also, compared to M82 the introgression sub-lines R176, R179, R181, and R202 showed a significant reduction of 27, 25, 18, and 27%, respectively. The FW was highly variable, ranging from 35 to 36 g (in R201 and R202) to 54–55 g (in R181 and R182), the latter values being not different from the parental genotype M82 (51.7 g). As for NFR, this value ranged from the minimum of 15.3 recorded in IL7-3 to the maximum of 32.4 recorded in R182, and no statistical differences were detected between the seven sub-lines and M82. Finally, as for the YP, only the genotype IL7-3 showed a value (0.60 kg/plant) significantly lower than M82 (1.58 kg/plant). In all the sub-lines a yield value similar to M82 was observed, with a mean of 1.27 kg/plant.

**Table 1:** Productivity-related traits (mean and standard deviation) of the seven sub-lines and their parental genotypes at Acerra in the year 2016.

Genotype	Flowers/ inflorescence (no.)	Fruit set (%)	Fruit weight (g)	Fruit/plant (no.)	Yield/plant (kg)
M82	5.1 ± 0.6	56.0 ± 5.0	51.7 ± 2.9	30.1 ± 7.9	1.58 ± 0.36
IL7-3	5.3 ± 0.7	35.6 ± 5.6^∗∗^	39.5 ± 1.8^∗∗^	15.3 ± 0.4^∗^	0.60 ± 0.03^∗∗^
R176	5.4 ± 0.5	40.9 ± 7.1^∗^	42.5 ± 4.0	24.7 ± 10.1	1.08 ± 0.12
R178	5.6 ± 0.6	53.4 ± 1.5	39.0 ± 6.6^∗^	30.9 ± 9.3	1.24 ± 0.56
R179	5.4 ± 0.3	41.7 ± 3.4^∗^	37.7 ± 2.3^∗∗^	27.7 ± 5.4	1.05 ± 0.17
R181	5.5 ± 0.7	46.0 ± 2.2^∗^	54.0 ± 8.9	26.9 ± 7.7	1.43 ± 0.34
R182	5.2 ± 1.5	53.9 ± 13.3	55.0 ± 1.0	32.4 ± 8.8	1.78 ± 0.28
R201	5.5 ± 0.7	51.5 ± 10.0	36.0 ± 1.7^∗∗^	29.1 ± 15.1	1.19 ± 0.34
R202	5.9 ± 0.6	40.9 ± 3.3^∗^	35.3 ± 3.5^∗∗^	29.0 ± 11.6	1.12 ± 0.25

*Data related to parameters linked to the production, such as the number of flowers per inflorescence and the fruit set, are reported together with those strictly related to the yield/plant, such as fruit weight and the number of fruit/plant. The significance of differences of each genotype vs. M82 was evaluated by the Student’s t-test (^∗^P < 0.05; ^∗∗^P < 0.01).*

The analysis of fruit quality traits carried out in the year 2016 (Table 2) showed levels significantly different between IL7-3 and M82. Among the analyzed traits, a significantly higher level was observed for °Brix in IL7-3 (5.77 °Brix) and in the sub-lines R178, R179, R182, R201, and R202 compared to M82 (3.87 °Brix). The fruit firmness (FI) was not different between M82 and IL7-3 (6.4 kg/cm^2^ for both), but four sub-lines showed levels of FI lower than the two parental lines, and only the sub-line R182 showed a very high level of FI (8.39 kg/cm^2^). The detection of fruit color in terms of a/b did not show significant differences between M82 and IL7-3, and all the sub-lines showed similar values, except than R176. Differences in the color were detected when measuring the chroma value, which showed a reduction of 12% in IL7-3 compared to the parental genotypes M82. The genotypes R178, R201, and R202 showed chroma values similar to IL7-3 (Table 2). Metabolic analysis carried out on mature fruit evidenced that IL7-3 accumulated significant higher levels of AsA compared to M82, confirming data collected in the past in our laboratory (Sacco et al., 2013; Calafiore et al., 2016). In particular, significantly higher AsA levels were observed in IL7-3 (39.88 mg/100 g FW) and in the sub-lines R182 (44.81 mg/100 g FW) and R202 (40.79 mg/100 g FW) compared to M82 (31.13 mg/100 g FW). No significant differences in total carotenoids, lycopene, and β-carotene content were observed between the two parental lines IL7-3 and M82, whereas the sub-lines R176, R178, R181, and R202 had reduced contents of total carotenoids and lycopene compared to M82 (Table 2).

**Table 2:** Fruit quality traits (mean and standard deviation) of the seven sub-lines and their parental genotypes at Acerra in the year 2016.

Genotype	Brix (°)	Firmness (kg/cm^2^)	Color (a/b)	Color (Chroma)	Total carotenoids (mg/100 g FW)	Lycopene (mg/100 g FW)	beta-carotene (mg/100 g FW)	AsA (mg/100 g FW)
M82	3.87 ± 0.25	6.48 ± 0.15	1.22 ± 0.09	41.92 ± 0.13	10.30 ± 0.92	6.76 ± 0.93	2.38 ± 0.79	31.13 ± 1.56
IL7-3	5.77 ± 0.55^∗∗^	6.42 ± 0.66	1.12 ± 0.09	37.10 ± 3.42^∗^	10.54 ± 2.09	6.31 ± 1.04	2.82 ± 0.27	39.88 ± 1.81^∗∗^
R176	4.37 ± 0.45	6.31 ± 0.13	1.04 ± 0.04^∗^	42.69 ± 0.54	5.75 ± 0.24^∗∗^	3.10 ± 0.15^∗∗^	1.33 ± 0.02	33.83 ± 0.84
R178	5.40 ± 0.72^∗^	5.31 ± 0.04^∗∗∗^	1.09 ± 0.09	37.65 ± 1.12^∗∗^	6.36 ± 0.28^∗∗^	3.50 ± 0.30^∗∗^	1.60 ± 0.14	38.54 ± 7.19
R179	4.73 ± 0.42 ^∗^	4.81 ± 0.02^∗∗∗^	1.18 ± 0.05	41.04 ± 3.29	7.66 ± 1.74	4.43 ± 1.13	1.60 ± 0.29	25.96 ± 2.66^∗^
R181	4.97 ± 1.07	5.48 ± 0.02^∗∗∗^	1.23 ± 0.03	43.50 ± 0.90^∗^	6.98 ± 1.41^∗^	4.06 ± 0.97^∗^	1.68 ± 0.26	32.44 ± 4.39
R182	4.97 ± 0.31^∗∗^	8.39 ± 0.62^∗∗^	1.29 ± 0.08	40.87 ± 0.95	9.22 ± 1.13	5.85 ± 0.13	2.14 ± 0.53	44.81 ± 2.31^∗∗^
R201	5.30 ± 0.26^∗∗^	6.04 ± 0.68	1.22 ± 0.12	37.75 ± 2.50^∗^	8.79 ± 2.07	5.17 ± 1.04	2.18 ± 0.26	37.55 ± 6.33
R202	5.47 ± 0.84^∗^	5.77 ± 0.14^∗∗^	1.20 ± 0.11	37.91 ± 0.37^∗∗∗^	7.19 ± 2.08	4.15 ± 0.99^∗^	1.91 ± 0.12	40.79 ± 3.73^∗^

*The significance of differences of each genotype vs. M82 was evaluated by the Student’s t-test (^∗^P < 0.05; ^∗∗^P < 0.01; ^∗∗∗^P < 0.001).*

**FIGURE 1:**
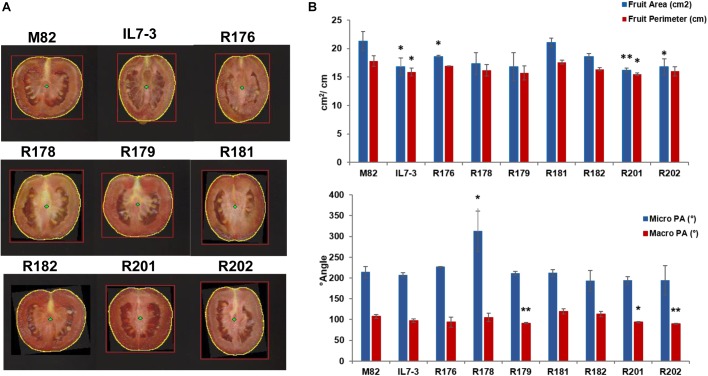
Morphology parameters measured on fruits of the seven sub-lines and their parental genotypes M82 and IL7-3 by the Tomato Analyzer software. **(A)** Longitudinal section images of the fruit with the yellow circle indicating the fruit perimeter (FP); **(B)** Graphs showing the values of FP and area (FA), and of micro and macro proximal angles (PA), and their significant differences from the control genotype M82 evaluated by the Student’s *t*-test (^∗^*P* < 0.05; ^∗∗^*P* < 0.01).

Finally, the morphological analysis of tomato fruits evidenced no significant differences for PA and PT (Supplementary Table S2 and Figure 1), and for the DA, whereas FP and FA were significantly reduced in IL7-3 and in few sub-lines (R176, R201, R202) compared to M82. In particular, a significant high correlation (*r* = 0.86, *p* = 0.0031) was detected between FA and FW. Also, the micro proximal angle (micro PA) and macro proximal angle (macro PA) were significantly different from M82 in some sub-lines (R178, R179, R201, R202). Only sub-lines R181 and R182 showed all morphological parameters similar to those exhibited by the control genotype M82.

**FIGURE 2:**
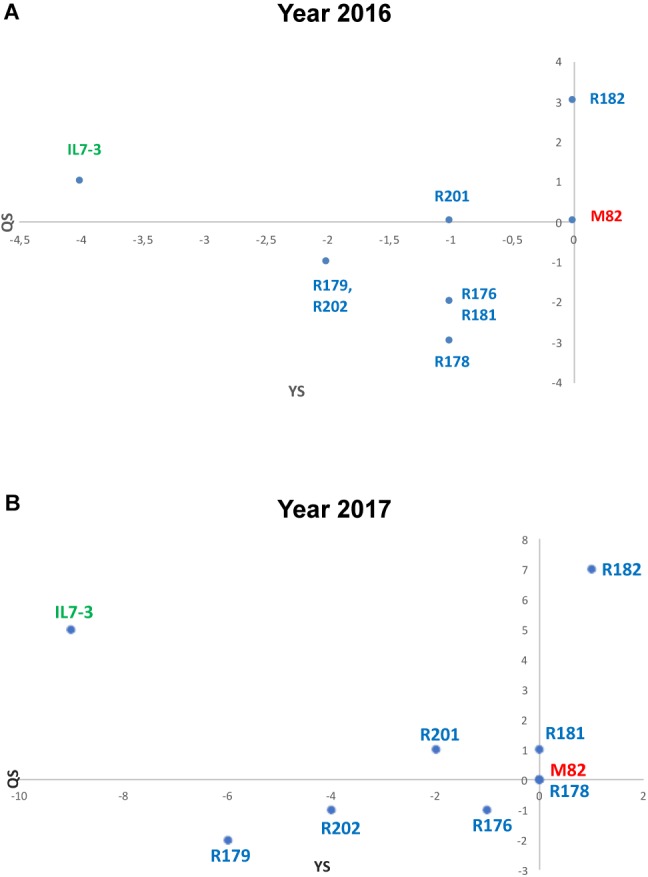
Evaluation Index (EI) estimated in the years 2016 **(A)** and 2017 **(B)** grouping data from productive (YS) and qualitative (QS) analyses. Data of the year 2016 derived from five yield-related scores and eight qualitative-related scores, whereas data from the year 2017 derived from three yield-related scores and three qualitative-related score measured in three experimental fields.

For a general selection of the sub-lines based on all evaluated traits, an EI was assayed, which takes into consideration the scores obtained by each line compared to the control genotype M82. The EI estimated for five productivity and eight fruit quality traits evidenced that the best sub-line in terms of quality and yield score (QS and YS, respectively) was R182, which exceeded the control genotype M82 for QS and had the same value for YS, as shown in the scatter diagram of Figure 2A. All the other sub-lines exhibited values of QS and YS worse than M82, and intermediate values between the two parental genotypes M82 and IL7-3.

### Multi-Environments Evaluation

In the summer of the year 2017 we restricted the analyses to the most relevant parameters among those measured in the year 2016, with the aim of evaluating them in three different environmental conditions in the South of Italy (Tables 3, 4). As for the parameters strictly concerning the productivity (FW, NFR, and YP), despite the differences observed in the data in the year 2017 in three different environments, no GxE interaction (univariate ANOVA test) was observed for FW (*p* = 0.657), as well as for NFR (*p* = 0.359) and YP (*p* = 0.256). The FW of the genotypes M82 and IL7-3 were significantly different in all the three fields. Differences of FW between the sub-lines and M82 were mainly observed in the Acerra field, where R178, R179, R201, and R202 exhibited a significant reduction compared to M82, confirming data obtained in the year 2016. In all the fields there was around a 50% NFR reduction of IL7-3 fruit compared to M82 (45.5%, 60.7%, 52.9% NFR reduction in Acerra, Giugliano and Battipaglia, respectively). Among the sub-lines, only R179 showed a significant reduction of NFR in all the fields, whereas R181, R182, R201, and R202 exhibited values comparable to M82 in the three environmental conditions tested. Finally, as for yield, IL7-3 showed a significant reduction with respect to M82 in all the fields, whereas the sub-lines R176, R178, R181, and R182 confirmed no differences compared to M82 in all the three trials.

Qualitative analyses carried out in 2017 were restricted to °Brix, FI, and AsA content in the ripe fruit, since these traits were the only one showing relevant differences among analyzed lines in the year 2016. As for °Brix, data collected in the year 2017 confirmed those of the year 2016; IL7-3 showed higher values than M82 in two fields out of three, evidencing a high GxE interaction (*p* = 0.008). Indeed, no differences were observed in Battipaglia, whereas in Acerra four introgression sub-lines showed significantly higher values than M82. As for firmness, the two parental genotypes and most sub-lines showed very similar values in all fields, with a not significant GxE interaction (*p* = 0.114). The only relevant result was the significantly higher firmness observed in R182 in all cases (values always higher than 8 kg/cm2). Finally, as for AsA, IL7-3 showed significantly higher values compared to M82 in the three fields, whereas most sub-lines showed a high variability, evidencing the presence of a GxE interaction (*p* = 0.042). Sub-line R182 was the only one showing a higher level of AsA content with respect to M82 in all the three experimental fields. The EI was also estimated on data collected in the year 2017 for the three production (FW, NFR, YP) and the three fruit quality traits (°Brix, FI, AsA) evaluated in three different environmental conditions. The scatter diagram (Figure 2B) evidences that the sub-line R182 exhibited the highest QS and YS, and that sub-lines R178 and R181 were comparable to M82. Therefore, in the second year of phenotypic analyses carried out in three different environmental conditions, the sub-line R182 was confirmed as the elite one. Finally, highly significant correlation coefficients were observed between all the traits evaluated in the Acerra field in the two years 2016 and 2017, except for firmness (Supplementary Table S3), thus confirming the robustness of our phenotypic analysis over the years.

**Table 3:** Productivity-related traits (mean and standard deviation) of the seven sub-lines and their parental genotypes in three different environments in the year 2017.

Genotype	Fruit weight (g)			Fruits/plant (no.)			Yield/plant (kg)		
	Acerra	Giugliano	Battipaglia	Acerra	Giugliano	Battipaglia	Acerra	Giugliano	Battipaglia
M82	51.2 ± 2.41	42.39 ± 2.49	60.28 ± 6.05	33.19 ± 2.69	60.76 ± 8.59	104.5 ± 19.5	1.7 ± 0.15	2.60 ± 0.73	3.36 ± 0.30
IL7-3	44.9 ± 2.52^∗^	36.78 ± 2.25^∗^	34.33 ± 1.7^∗∗^	17.89 ± 2.48^∗∗^	24.00 ± 1.65^∗∗^	48.6 ± 11^∗^	1.07 ± 0.10^∗∗^	1.30 ± 0.27^∗^	1.17 ± 0.55^∗∗^
R176	47.6 ± 21.68	49.02 ± 7.37	51.58 ± 18.63	31.97 ± 1.26	38.67 ± 5.83^∗^	117.0 ± 57.1	1.67 ± 0.58	2.65 ± 0.93	3.15 ± 1.12
R178	40.3 ± 5.67^∗^	36.8 ± 6.32	42.71 ± 12.15	42.56 ± 3.75^∗^	58.42 ± 7.70	102.6 ± 53.2	1.71 ± 0.21	2.63 ± 0.32	2.87 ± 0.97
R179	43.3 ± 2.67^∗^	39.3 ± 5.56	53.95 ± 20.07	23.74 ± 4.99^∗^	38.86 ± 7.28^∗^	42.1 ± 28.5^∗^	1.03 ± 0.23^∗^	2.03 ± 0.33	1.12 ± 0.39^∗∗^
R181	56.4 ± 6.30	45.6 ± 9.63	59.74 ± 5.72	32.14 ± 1.03	42.93 ± 15.83	106.5 ± 41.7	1.81 ± 0.21	2.33 ± 0.61	3.82 ± 1.60
R182	51.6 ± 5.35	50.3 ± 2.25^∗^	58.03 ± 9.43	36.54 ± 12.55	54.29 ± 10.94	114.3 ± 38.4	1.87 ± 0.63	2.70 ± 1.23	3.51 ± 0.47
R201	39.6 ± 3.02^∗∗^	32.6 ± 7.24	44.67 ± 12.68	38.18 ± 4.93	50.31 ± 18.56	84.1 ± 25.9	1.52 ± 0.30	2.51 ± 1.01	1.99 ± 0.45^∗^
R202	40.3 ± 1.06^∗∗^	37.3 ± 5.21	46.07 ± 4.93^∗^	35.40 ± 1.60	41.58 ± 14.00	81.2 ± 12.3	1.43 ± 0.07^∗^	2.11 ± 0.74	2.06 ± 0.53^∗^

		**F**	**Sign**		**F**	**Sign**		**F**	**Sign**

ENVIRONMENT		9.504	0.000		49.952	0.000		17.314	0.000
GENOTYPE		6.297	0.000		3.760	0.001		6.261	0.000
GxE		0.821	0.657		1.123	0.359		1.261	0.256

*The ANOVA test results are reported for each trait as F value, to estimate the environment, the genotype and their interaction effect, considering as level of significance a < 0.05. The significance of differences of each genotype vs. M82 was evaluated by the Student’s t- test (^∗^P < 0.05; ^∗∗^P < 0.01).*

**Table 4:** Fruit quality traits (mean and standard deviation) of the seven sub-lines and their parental genotypes in three different environments in the year 2017.

Genotype	°Brix			Firmness (kg/cm^2^)			AsA (mg/100 g FW)		
	Acerra	Giugliano	Battipaglia	Acerra	Giugliano	Battipaglia	Acerra	Giugliano	Battipaglia
M82	4.60 ± 0.20	4.07 ± 0.57	4.97 ± 0.47	7.07 ± 0.52	6.49 ± 0.15	7.34 ± 0.39	44.35 ± 6.55	27.38 ± 2.04	26.10 ± 3.24
IL7-3	6.47 ± 0.32^∗∗^	6.00 ± 0.26^∗∗^	5.30 ± 0.36	6.52 ± 0.88	6.42 ± 0.66	6.38 ± 0.82	64.21 ± 5.87^∗^	38.17 ± 3.08^∗∗^	40.87 ± 3.70^∗∗^
R176	4.90 ± 0.26	4.40 ± 0.46	4.57 ± 0.15	7.73 ± 0.90	6.27 ± 0.15	6.95 ± 0.75	39.90 ± 6.53	16.51 ± 5.36^∗^	22.26 ± 2.80
R178	5.93 ± 0.47^∗^	5.40 ± 0.72	4.73 ± 0.35	5.98 ± 0.75	5.47 ± 0.57^∗^	6.64 ± 1.02	51.75 ± 9.90	24.63 ± 0.40	26.96 ± 4.98
R179	5.13 ± 0.85	4.73 ± 0.42	5.30 ± 0.53	6.70 ± 1.43	4.81 ± 0.33^∗∗^	7.38 ± 0.39	43.91 ± 8.97	31.13 ± 9.67	15.10 ± 5.88^∗^
R181	5.10 ± 0.17^∗^	4.97 ± 1.07	4.87 ± 0.51	8.46 ± 0.81	7.16 ± 1.50	6.46 ± 0.85	49.67 ± 2.96	35.81 ± 8.45	30.34 ± 3.97
R182	5.93 ± 0.55^∗^	4.96 ± 0.31	3.93 ± 0.38	8.32 ± 0.57^∗^	8.29 ± 0.54^∗∗^	8.77 ± 0.53^∗^	57.86 ± 3.77^∗^	34.82 ± 2.21^∗^	34.34 ± 1.24^∗^
R201	4.97 ± 0.15	5.30 ± 0.26^∗^	5.40 ± 0.87	6.53 ± 0.39	6.12 ± 0.56	5.94 ± 0.82^∗^	46.87 ± 3.17	35.93 ± 3.31^∗^	25.56 ± 4.32
R202	5.63 ± 0.38^∗^	5.47 ± 0.84	4.70 ± 0.35	5.86 ± 0.53^∗^	6.06 ± 0.21^∗^	6.98 ± 0.76	55.26 ± 6.47	33.08 ± 4.40	25.53 ± 1.18

		**F**	**Sign**		**F**	**Sign**		**F**	**Sign**

ENVIRONMENT		8.039	0.000		4.943	0.000		142.446	0.000
GENOTYPE		5.882	0.001		8.500	0.011		13.611	0.000
GxE		2.413	0.008^∗∗^		1.555	0.114		1.905	0.042^∗^

*The ANOVA test results are reported for each trait as F value, to estimate the environment, the genotype and their interaction effect, considering as level of significance a < 0.05. The significance of differences of each genotype vs. M82 was evaluated by the Student’s t- test (^∗^P < 0.05; ^∗∗^P < 0.01).*

### Molecular Marker Analysis

In order to better define the size of *S. pennellii* introgression region in the selected sub-line R182, 15 SCAR, and three CAPS molecular markers were designed on some of the genes mapping on the introgressed region (Supplementary Table S4). As a whole, these markers targeted 14 out of 32 genes mapping in the wild region of R182, considering that in four cases (Solyc07g047990, Solyc07g048010, Solyc07g049140, Solyc07g049310) two markers were designed for each gene (Table 5). The cultivated region covered by the designed molecular markers spans from 59,320,014 to 59705558 bp of chromosome 7), whereas the corresponding *S. pennellii* region spans from 69831629 to 70130606 bp. All markers were constructed based on polymorphisms found comparing the genomes of the cultivated and the wild species and were tested on the parental genotypes M82 and IL7-3 together with R182. This analysis allowed determining whether the wild or the cultivated allele is present for each investigated gene in the introgressed region of the sub-line R182, thus precisely defining its borders. As reported in Table 5, the region spanning from marker MK2 to marker MK21 carries wild alleles in the sub-line R182, since R182 showed the same amplified or digested DNA fragments observed in the parental line IL7-3. The last wild gene of the introgressed region is Sopen07g024640, for which two different SCAR markers were designed (MK21 and MK22), targeting different regions of the gene. Since the genotype R182 showed the wild allele for marker MK21 and the cultivated one for marker MK22, we hypothesized that the recombination event leading to the definition of the introgression region in this line may have occurred within this gene.

**Table 5:** Polymorphisms evidenced in the introgression region of the sub-line R182 by screening 18 molecular markers to determine the size of the region. (+): *S. pennellii* allele; (−): *S. lycopersicum* allele.

Marker code^1^	*S. lycopersicum* gene targeted	Chr. 7 start position SL3.0	S. *pennellii* gene targeted	Chr. 7 start position *S.pennellii*	M82	IL7-3	R182
MK1	Solyc07g047980	59320014	Sopen07g024410	69831629	−	−	−
MK2	Solyc07g047990	59325229	Sopen07g024420	69836399	−	+	+
MK3	Solyc07g047990	59325229	Sopen07g024420	63836805	−	+	+
MK4	Solyc07g048000	59330722	Sopen07g024430	69840798	−	+	+
MK5	Solyc07g048010	59335411	Sopen07g024440	69849442	−	+	+
N34	Solyc07g048010	59338020	Sopen07g024440	69848612	−	+	+
MK6	Solyc07g048030	59347798	Sopen07g024450	69859998	−	+	+
MK8	Solyc07g048080	59394940	Sopen07g024500	69913622	−	+	+
MK9	Solyc07g048100	59477649	Sopen07g024530	69961500	−	+	+
MK11	Solyc07g049140	59494927	Sopen07g024560	69998524	−	+	+
MK12	Solyc07g049140	59494979	Sopen07g024560	69998568	−	+	+
MK13	Solyc07g049170	59554063	Sopen07g025160	70804301	−	+	+
MK14	Solyc07g049200	59621820	Sopen07g025210	70873975	−	+	+
N35	Solyc07g049280	59667971	Sopen07g024610	70081676	−	+	+
MK20	Solyc07g049290	59670170	Sopen07g024620	70082413	−	+	+
MK21	Solyc07g049310	59686924	Sopen07g024640	70111820	−	+	+
MK22	Solyc07g049310	59695121	Sopen07g024640	70120070	−	+	−
N18	Solyc07g049320	59705558	Sopen07g024650	70130606	−	+	−

*Marker code: MKx = SCAR markers; Nx = CAPS markers.*

## Discussion

In the present work, seven introgression sub-lines from the *S. pennellii* in *S. lycopersicum* IL7-3 were characterized in different years and in different environmental conditions. These lines were previously selected in our laboratory at the Department of Agricultural Sciences at the University of Naples Federico II by combining genomics tools with metabolic analyses, with the aim of identifying QTLs and candidate genes mapping in the introgression regions that could be involved in the control of nutritional quality traits (Calafiore et al., 2016). Additional information related to fruit morphology, yield-related parameters and fruit quality traits where obtained in the present work. One of the most critical aspects of growing the IL7-3 is its lower productivity compared to M82 (Eshed and Zamir, 1995; Sacco et al., 2013). Therefore, a specific investigation on the NFL, FS, NFR and FW was carried out in this paper in all the lines. In IL7-3 compared to M82 a lower FS and also a reduced number of fruits was observed, which together with a significant lower FW, contributed to the reduced yield of the IL. On the contrary, a similar yield value was observed in almost all the introgression sub-lines when compared to M82, except than R179 and R202, which exhibited a significant yield reduction in at least two experimental fields out of four (one trial in the year 2016 and three trials in the year 2017). In the first genotype this yield reduction is mainly due to a reduction of NFR, whereas in R202 it seems linked to a reduction in FW. These results are in accordance with data previously reported in tomato, which suggested that both traits (FW and NFR) are relevant yield components to be used to select tomato genotypes with higher yield (Monamodi, 2013; Gur and Zamir, 2015). By contrast, for all yield-related traits the lines R181 and R182 showed stable good performances in all the environments tested.

**FIGURE 3:**
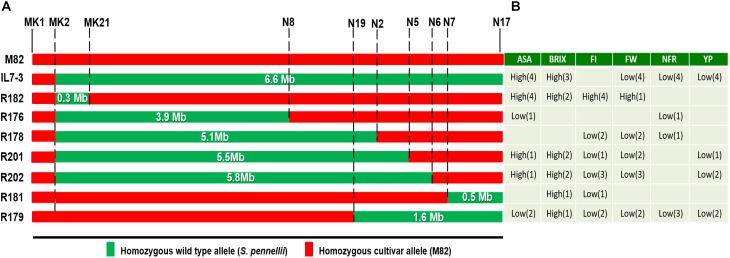
Effect of the wild genome on productive and qualitative traits evaluated on the sub-lines of IL7-3. **(A)** Introgression region size (in Mbp) from *S. pennellii* on chromosome 7 of IL7-3 and of the seven sub-lines; markers coded N (reported in Calafiore et al., 2016) targeting the borders of each introgression are reported; markers coded MK define the borders of R182 sub-line; **(B)** Values of analyzed traits that were significantly higher (high) or lower (low) than M82 (in parenthesis is reported the number of experimental trials where these values were observed). Figure modified from Calafiore et al. (2016).

As for the quality traits analyzed, most sub-lines exhibited high levels of °Brix and AsA, thus resembling IL7-3, with values often exceeding those usually observed in current tomato cultivars. For example, a vitamin C content ranging between 15 and 21 mg/100 g FW was reported by Abushita et al. (2000) in salad tomatoes grown in field conditions, whereas a mean value of 19 mg/100 g FW in processing tomatoes. Frusciante et al. (2007) reported a vitamin C content between 8.0 and 16.3 mg/100 g FW, and Ruggieri et al. (2016) values of AsA ranging from 10 to 40 mg/100 g of FW in commercial varieties. Therefore, the values over 35 mg/100 g FW usually observed in the mature fruit of R182 in our experiments are high enough for the improvement of current varieties. As a whole, the best two lines were R181 and R182, since they combined good quality traits with a yield comparable to the control genotype M82 and with small wild introgressed regions (506 and 385 Kbp, respectively; Figure 3). However, only R182 exhibited a stable higher AsA content compared to M82 over the trials conducted. In addition, R182 exhibited a surprisingly high firmness when evaluated in different environmental conditions, even though such a high level of firmness was not observed in the parental lines. For all the other evaluated traits, including fruit morphology, the sub-line R182 performed as the control genotype M82. The potentially higher nutritional value of R182 fruits combined with the other good traits makes the sub-line R182 the best one selected in the present work, as also evidenced by the EI values estimated in both years 2016 and 2017.

Considering the introgression regions of all the sub-lines analyzed (Figure 3), the two sub-lines R181 and R182 carry the smallest wild segments, but in opposite positions. Therefore, probably the genes that negatively affect yield map in the central part of IL7-3 introgression region, and mainly control FW and NFR, whereas the gene/s stably increasing the AsA content in the fruit is/are located in the upper part of the introgression region (left part in Figure 3). Moreover, the unexpected high firmness observed in the sub-line R182 may be due to new interactions between genes and/or regulatory elements mapping in the wild region of R182 and in the nearby cultivated regions, originated by the specific recombination events that occurred to generate this sub-line. These new positive interactions have probably contributed also to obtain the very stable high accumulation of AsA in the fruit of R182, as evidenced by data collected over the years and over the different experimental fields. Alternatively, the occurrence of unexpected traits in the line R182 can be also explained by the presence of smaller genomic regions coming from the wild *S. pennellii* or from other *S. lycopersicum* varieties due to cross contaminations. Therefore, the background genotype of R182 is being further investigated by GBS and RNAseq analyses to assure that this sub-line is really isogenic. From data obtained by these two deep-sequencing approaches it will be verified if the novel traits recorded in the sub-line R182 could be ascribed also to other regions actually unknown.

The positive performances observed in the sub-line R182 for the quality and agronomic traits studied could be due to the activity of genes/regulatory factors/elements mapping in the small wild region (385 Kbp) present in this introgression sub-line. The effect on such a different number of traits indicates the great potential of this small introgressed region on chromosome 7. For this reason, the presence of wild/cultivated alleles mapping in this introgressed region was investigated by designing gene-targeting markers, based on the sequencing information deriving from the whole genome of *S. lycopersicum* (The Tomato Genome Consortium, 2012) and *S. pennellii* (Bolger et al., 2014), both available in the Sol Genomics Network database^2^. In our previous work the introgression region size of the sub-line R182 was estimated by CAPS markers as covering approximately 305 Kb (from marker N27 at 59,218,000 bp to marker N14 at 59,523,504 bp of SL2.5 version of chromosome 7) (Calafiore et al., 2016). In the meanwhile, a new annotation of the tomato genome was released in the year 2017 (SL3.0) and therefore we designed additional markers covering in more details this introgressed region. In particular, we constructed SCAR markers in the upper part of the introgressed region 7-3, since this includes wild genes carried by the sub-line R182, as evidenced from the species-specific CAPS markers previously analyzed. Based on a RNA-seq study carried out on the *S. pennellii* ILs to understand the genetic basis for leaf morphology (Chitwood et al., 2013), it was established that the wild introgressed region of IL7-3 starts from the gene Solyc07g047990 and stops at the gene Solyc07g063390. The study carried out in this paper demonstrated that the sub-line R182 carries wild alleles from the gene Solyc07g047990, ortholog of Sopen07g024420, to the gene Solyc07g049310 ortholog of Sopen07g024640. This wild region has an estimated size of 385 Kbp and includes around 32 genes in the wild genome, which replaced 36 genes of the cultivated one (Supplementary Table S5). Overall, these wild genes should positively affect quality traits, such as AsA and firmness, without detrimental effects on yield.

Among the genes mapping in the *S. pennellii* introgressed region in R182, a Pyrophosphate-fructose 6-phosphate 1-phosphotransferase (PFP) subunit beta (encoded by the gene Solyc07g049280 corresponding to the *S. pennellii* ortholog Sopen07g024610) and two Major facilitator superfamily (MFS) proteins (Solyc07g049290/Sopen07g024620 and Solyc07g049310/Sopen07g024640) were identified. These genes could have a role in sugar accumulation affecting °Brix and indirectly also AsA content, being sugars precursors of its biosynthesis (Di Matteo et al., 2010). In particular, PFP is a key enzyme in the Glycolysis/Gluconeogenesis pathways, which catalyzes the reversible interconversion between fructose-6-phosphate and fructose-1, 6-bisphosphate (Duan et al., 2016). MFSs are membrane proteins involved in the import or export of sugars, especially during fruit ripening (Reuscher et al., 2014). The expression profile (data retrieved from Tomato Expression Atlas^2^ and TomExpress^3^) of these genes, in particular Solyc07g049290, exhibits a typical ripening pattern, with expression increasing until the breaker stage and then decreasing at red ripe stage, indicating as these genes could be active players in processes linked to sugar and AsA accumulation in the fruit.

Regarding firmness, three genes map in the introgressed region of R182, which are potentially involved in the control of this trait. Genes Solyc07g048085 and Solyc07g048090 encode for the cell wall proteins Fasciclin-like arabinogalactan and the gene Solyc07g049300 encodes for a polygalacturonase, which is a protein potentially involved in cell wall degradation (Huang et al., 2017). Further work will be carried out in order to identify the exact function of all the identified candidate genes through functional genomic approaches by using techniques such as the CRISPR/Cas9 technology to produce knock-out mutants.

High-resolution fine mapping investigations in tomato ILs populations were previously carried out to identify loci controlling fruit qualitative traits (Liu et al., 2016). For example, the authors identified a small segment of 200 Kbp altering the content of different metabolites by fine-mapping a number of sub-lines of the IL4-4. Herein, we have identified a group of around 32 genes mapping in a wild *pennellii* region of 385 Kbp, which exhibit a broad positive effect on nutritional quality and other traits. This result demonstrates that exploiting genetic variation deriving from a wild species could be extremely useful to improve the cultivated tomato, and also that this variation could be rapidly available to plant breeders, since no traits were negatively affected by the wild genomic segment introgressed in the sub-line R182.

## Author Contributions

RC contributed to metabolic and molecular markers analyses. VR contributed to bioinformatics analysis of the introgressed region. RC and VR drafted the manuscript. AA and FO contributed to growing and evaluating the materials in the fields and in laboratory. MR contributed to the conception of the experiment and critically reviewed the manuscript. AB contributed to the experiment design, to data analysis and interpretation, to draft the manuscript.

## Conflict of Interest Statement

The authors declare that the research was conducted in the absence of any commercial or financial relationships that could be construed as a potential conflict of interest.

## Footnotes

1 https://vanderknaaplab.uga.edu/

2 http://solgenomics.net

3 http://tomexpress.toulouse.inra.fr/
