# Supplementary material for: Phenotypic and Molecular Selection of a Superior Solanum pennellii Introgression Sub-Line Suitable for Improving Quality Traits of Cultivated Tomatoes
Source: Front Plant Sci. 2019 Feb 22;10:190. doi: 10.3389/fpls.2019.00190 (PMC6395448; doi:10.3389/fpls.2019.00190)
Supplement: Supplementary file 2 [file Table_2.docx]

| **Genotype** | **FA (cm^2^)** | **FP (cm)** | **PA (cm2)** | **PT (cm)** | **DA (°)** | **Micro PA (°)** | **Macro PA (°)** |
| --- | --- | --- | --- | --- | --- | --- | --- |
| **M82** | 21.32±1.67 | 17.81±0.90 | 0.42±0.02 | 0.36±0.02 | 169.56±7.18 | 213.84 ±14.00 | 107.50±5.15 |
| **IL7-3** | 16.77±1.55* | 15.87±0.70* | 0.40±0.03 | 0.38±0.03 | 171.81±2.08 | 207.22±5.27 | 97.21±3.85 |
| **R176** | 18.62±0.16* | 16.94±0.05 | 0.43±0.03 | 0.34±0.03 | 170.88±7.18 | 226.55±1.35 | 93.66±11.71 |
| **R178** | 17.35±1.91 | 16.19±1.03 | 0.42±0.05 | 0.36±0.04 | 169.93±2.68 | 312.74±48.79* | 104.83±10.05 |
| **R179** | 16.80±2.45 | 15.71±1.26 | 0.41±0.03 | 0.36±0.03 | 168.48±3.71 | 210.99±4.89 | 91.32±1.39** |
| **R181** | 21.08±0.76 | 17.59±0.36 | 0.45±0.04 | 0.33±0.03 | 173.50±6.64 | 212.59±8.10 | 119.95±6.45 |
| **R182** | 18.61±0.53 | 16.37±0.30 | 0.44±0.01 | 0.34±0.01 | 168.48±3.35 | 193.28±25.06 | 113.47±6.19 |
| **R201** | 16.21±0.36** | 15.50±0.21* | 0.40±0.02 | 0.38±0.01 | 175.28±4.42 | 194.57±8.84 | 93.76±1.91* |
| **R202** | 16.78±1.41* | 16.00±0.81 | 0.40±0.04 | 0.37±0.02 | 170.22±0.08 | 194.36±35.77 | 90.13±0.84** |

**Supplementary Table S2**- Morphological traits measured by the Tomato Analyzer software on the seven sub-lines (coded R) and their parental genotypes M82 and IL7-3. The significance of differences of each genotype *vs* M82 was evaluated by the Student’s t-test (*: P<0.05; **: P<0.01;).

FA=Fruit area; FP=Fruit perimeter; PA=Pericarp Area; PT=Pericarp thickness; DA=Distal angle; Micro and Macro PA= Micro and Macro Proximal angle
