# Supplementary material for: Phenotypic and Molecular Selection of a Superior Solanum pennellii Introgression Sub-Line Suitable for Improving Quality Traits of Cultivated Tomatoes
Source: Front Plant Sci. 2019 Feb 22;10:190. doi: 10.3389/fpls.2019.00190 (PMC6395448; doi:10.3389/fpls.2019.00190)
Supplement: Supplementary file 3 [file Table_3.DOCX]

**Supplementary Table S3** – Correlation coefficients (r) and their significance level (p) between traits evaluated at Acerra in the years 2016 and 2017.

| **Trait** | **r** | **p** |
| --- | --- | --- |
| YP | 0.795 | 0.0104 |
| NFR | 0.811 | 0.0079 |
| FW | 0.936 | 0.0002 |
| Brix | 0.793 | 0.0107 |
| FI | 0.480 | 0.1908 |
| AsA | 0.734 | 0.0245 |
